# Pediatric cataract, myopic astigmatism, familial exudative vitreoretinopathy and primary open-angle glaucoma co-segregating in a family

**Published:** 2011-08-10

**Authors:** D.A. Mackey, A.W. Hewitt, J.B. Ruddle, B. Vote, R.G. Buttery, C. Toomes, R. Metlapally, Y.J. Li, K.N. Tran-Viet, F. Malecaze, P. Calvas, T. Rosenberg, J.A. Guggenheim, T.L. Young

**Affiliations:** 1 Centre for Ophthalmology and Visual Science, University of Western Australia, Lions Eye Institute, Perth, Australia; 2 Centre for Eye Research Australia, University of Melbourne, Department of Ophthalmology, Royal Victorian Eye and Ear Hospital, Melbourne, Australia; 3 Vitreoretinal Unit, Royal Victorian Eye and Ear Hospital, Melbourne, Australia; 4 Eye Department, University of Tasmania, Royal Hobart Hospital, Hobart, Australia; 5 The Launceston Eye Institute, Launceston, Australia; 6 Section of Ophthalmology and Neuroscience, Leeds Institute of Molecular Medicine, University of Leeds, Leeds, UK; 7 Department of Ophthalmology, Duke University Eye Center, Durham, NC; 8 School of Optometry, University of California at Berkeley, Berkeley, CA; 9 Department of Biostatistics and Bioinformatics, Duke University Medical Center, Durham, NC; 10 Center for Human Genetics, Duke University Medical Center, Durham, NC; 11 Toulouse University Hospital, Université Paul Sabatier, Toulouse, France; 12 Kennedy Center, Glostrup, Denmark; 13 School of Optometry and Vision Sciences, Cardiff University, Cardiff, Wales, United Kingdom

## Abstract

**Purpose:**

To describe an Australian pedigree of European descent with a variable autosomal dominant phenotype of: pediatric cortical cataract (CC), asymmetric myopia with astigmatism, familial exudative vitreoretinopathy (FEVR), and primary open-angle glaucoma (POAG).

**Methods:**

Probands with CC, FEVR, and POAG were enrolled in three independent genetic eye studies in Tasmania. Genealogy confirmed these individuals were closely related and subsequent examination revealed 11 other family members with some or all of the associated disorders.

**Results:**

Twelve individuals had CC thought to be of childhood onset, with one child demonstrating progressive lenticular opacification. One individual had severe retinal detachment while five others had dragged retinal vessels. Seven individuals had POAG. Seven individuals had myopia in at least one eye ≤-3 Diopters. DNA testing excluded mutations in myocilin, trabecular meshwork inducible glucocorticoid response (*MYOC*) and tetraspanin 12 (*TSPAN12*). Haplotype analysis excluded frizzled family receptor 4 (*FZD4*) and low density lipoprotein receptor-related protein 5 (*LRP5*), but only partly excluded *EVR3*. Multipoint linkage analysis revealed multiple chromosomal single-nucleotide polymorphisms (SNPs) of interest, but no statistically significant focal localization.

**Conclusions:**

This unusual clustering of ophthalmic diseases suggests a possible single genetic cause for an apparently new cataract syndrome. This family’s clinical ocular features may reflect the interplay between retinal disease with lenticular changes and axial length in the development of myopia and glaucoma.

## Introduction

In this study, we describe the novel overlapping phenotype of congenital cataract (CC), familial exudative vitreoretinopathy (FEVR), myopia, and primary open-angle glaucoma (POAG) segregating in an apparently autosomal-dominant fashion.

In Australia, myopia affects approximately 15% of the population [1], POAG affects approximately 3% of the population [2], CC occurs in approximately 2.2 out of every 10,000 births [3], and FEVR affects an estimated 7 out of every 1000,000 people (derived from comparing 13 indexed FEVR cases [4] to 420 CC cases [3]). If we were to consider these diseases as completely independent clinical entities, the highly unlikely probability of a patient having all four diseases simultaneously, or of the four diseases co-segregating, would be approximately 1 in 148 billion. This denominator is more than 20 times the total population of earth today.

Interestingly, to some extent these clinical entities can be associated with each other. Many investigators have reported the association of high myopia with cataract, glaucoma, and retinal detachment [5]. Other associations are less common:

•anterior polar cataracts, seen in aniridia, are often associated with glaucoma [6];
•rubella embryopathy is associated with both congenital glaucoma and CC [6];
•aphakic glaucoma is observed very frequently, and cataract can develop as a complication of POAG-filtering surgery [6];
•retinal detachment is a feature of Stickler syndrome and is associated often with cortical lens opacities [7];
•retinal detachment from retinopathy of prematurity (ROP) is associated with myopia and cataract [8].
•Retinal dystrophies are associated with myopia and posterior subcapsular cataracts [9].

Although researchers have identified genes associated with each of these disorders, the genetic mechanisms and their interactions still are not fully understood.

## Methods

We identified three closely-related index cases from three genetic-eye-disease studies: VI:7 from the Glaucoma Inheritance Study in Tasmania (GIST) [10], VIII:7 from the Cataract Inheritance Study in South Eastern Australia (CISSEA) [3], and VIII:8 from the Familial Retinal Detachment Study (FRDA) [4]. The GIST study had ethical approval from the Royal Hobart Hospital; the CISSEA and FRDA studies had ethical approval from the Royal Victorian Eye and Ear Hospital. In each case, the work was conducted in accordance with the tenets of the Declaration of Helsinki.

When we realized that the index cases were a grandmother and two of her grandchildren who were genetic first cousins, we decided to examine the entire pedigree in detail to characterize a potentially novel phenotype. Our ultimate aim was to identify the gene responsible for this apparently-autosomal-dominant disorder.

From the genealogy of the index cases [11] we identified the living members of five lineal generations, as well as surviving more-distant relatives. We invited these family members for a comprehensive ophthalmic examination [12], including:

•a LogMAR visual acuity test,
•the Goldmann applanation intraocular pressure (IOP) measurement,
•refraction using a HARK-598 autorefractor (Carl Zeiss Meditec, Miami, FL),
•axial length measurement using an Ocuscan^®^ (Alcon, Inc., Ft Worth, TX),
•corneal pachymetry using an IOPac (Heidelberg Instruments, Heidelberg, Germany),
•lens photographs,
•stereoscopic optic disc photography using a Nidek 3Dx camera (Nidek, Gamagori, Japan), and
•examination of the peripheral retina.

All participants provided venous blood or saliva specimens for DNA extraction and genetic analysis.

Genotyping was performed using fluorescently-tagged microsatellite markers as described previously [13]. Briefly, standard PCR reactions were carried out in a 25 μl volume containing 50 ng of genomic DNA using Invitrogen Taq DNA polymerase and buffers (Invitrogen). Microsatellite markers (including primer details; Table 1) surrounding EVR1 (D11S4187, D11S896, and D11S1367), EVR4 (D11S2006, D11S4095, and D11S937) and EVR3 (D11S929, D11S4115, D11S4154, D11S4203, D11S4083, and D11S4102) were selected from the genome browser. Following amplification, PCR products were resolved using an ABI 3730 DNA sequencer and analyzed using GeneMapper^®^ software from the same manufacturer (Applied Biosystems, Carlsbad, CA). The coding sequence and surrounding exons of myocilin, trabecular meshwork inducible glucocorticoid response (*MYOC*) and tetraspanin 12 (*TSPAN12*; primers and conditions are listed in Table 2) were screened using standard direct sequencing protocols as described previously (see above) [14,15].

**Table 1:** Microsatellite primers and conditions.

**Marker**	**Primer names and sequences (5’-3’)**	**Size (bp)**	**Annealing temperature**	**Amplification conditions**
D11S4187	F TCTTGAACCCGGGAAG	273-289	55 °C	Invitrogen Taq & buffer
	R CTGGTGCTGTGCTTGG			
D11S896	F ATCTCCCCTAGCTGTTTTGGA	169-183	60 °C	Invitrogen Taq & buffer
	R AGTTCATATCCACCTCACACA			
D11S1367	F GCTGACATTTATTCACATGGC	224-244	60 °C	Invitrogen Taq & buffer
	R ACAGTGTTATCTCCCTGGCA			
D11S2006	F CTTGTGGGCTGTAGTTTGCT	~325	55 °C	Invitrogen Taq & buffer
	R AAAGAGTAAACTCAATGAAAGATGC			
D11S4095	F TCCCTGGCTATCTTGAATC	173-205	55 °C	Invitrogen Taq & buffer
	R CTTGACTGGGTCCACG			
D11S937	F CTAATAAACAAATCCCTCTACCTCC	230-264	60 °C	Invitrogen Taq & buffer
	R TAGTCAGTCAGGGACCCAAGT			
D11S929	F AGGCCCTTCCAAGATCAG	218-240	60 °C	Invitrogen Taq & buffer
	R CCCAGTTGCCGAACTACC			
D11S4115	F TGGCATGTAAATNTAAGAGACTCAC	185-199	50 °C	Invitrogen Taq & buffer
	R CTGCTACCTCAGAAGTATCTCAA			
D11S4154	F ATCCCTTGGCTTTCTCAGAGCAC	146-158	65 °C	Invitrogen Taq & buffer
	R GGTGCCCCTAACCTCCATGT			
D11S4203	F GAATAGCCACTGACTTCAGG	218-278	60 °C	Invitrogen Taq & buffer
	R CAGGATGCTGGAATAGAGAA			
D11S4083	F TTTAACCCAAGGGCAGGAC	178-206	55 °C	Invitrogen Taq & buffer
	R CATGTGTACCCAAGGGCAG			
D11S4102	F CACCACTGGGTACTGCCATC	142-174	60 °C	Invitrogen Taq & buffer
	R GCTAAATCCTGGAAAGCCCTG			

**Table 2:** *TSPAN12* primers and PCR conditions.

**Exon**	**Primer names and sequences (5’-3’)**	**Size (bp)**	**Annealing temperature**	**Amplification conditions**
2	TSPAN12-ex2-F ATGTCCCGTGTTCTCTCTCC	382	60 °C	Invitrogen Taq & buffer
	TSPAN12-ex2-R CCAGGGGTGGATTTCTTTGT			
3	TSPAN12-ex3-F TGGTAATTGGGAAAGATATTATGTAAC	291	60 °C	Invitrogen Taq & buffer
	TSPAN12-ex3-R CCAAAAGATCAAGGAAGAGCA			
4	TSPAN12-ex4-F TGAGGCATCATGATTGAAAGAA	346	60 °C	Invitrogen Taq & buffer
	TSPAN12-ex4-R GCTATCACTGCTCCCTAATCTTGT			
5	TSPAN12-ex5-F GGTCCCCTTTCTTGGAGAAC	947	60 °C	Invitrogen Taq & buffer
	TSPAN12-ex5-R TGGAAATGTGCTTTAGACACAGA			
6	TSPAN12-ex6-F GTACAAAATACCTCTTCATTTATCACA	529	60 °C	Hot shot master mix
	TSPAN12-ex6-R GAAGAAAAGCAGGCCATGAA			
7	TSPAN12-ex7-F TGATGACAGATATAGCTCTGGGT	376	60 °C	Hot shot master mix
	TSPAN12-ex7-R TTTTAAGGCCTTTTACATTTAGACA			
8	TSPAN12-ex8-F GCTTTCCCTGAGAACCACTG	605	60 °C	Hot shot master mix
	TSPAN12-ex8-R CCATCCTCATTTTAAAGCATAGA			

For the genotyping platform, we used Linkage Panel IVb of 6008 genome-wide single-nucleotide polymorphisms **(**SNPs; Illumina, San Diego, CA), and ran the analysis at the Center for Inherited Disease Research (CIDR) of Johns Hopkins University (Baltimore, MD). The results for the pedigree were analyzed with Fastlink using a 2-point analysis (under a dominant model); multipoint results (both parametric and non-parametric) were analyzed using MERLIN. Merlin (Multipoint Engine for Rapid Likelihood Inference) is a software package that uses sparse inheritance trees for pedigree analysis [16].

## Results

Genealogical information was available for nine generations of the participants’ family; the individuals examined for this study came from the five most recent generations.

**Figure 1:**
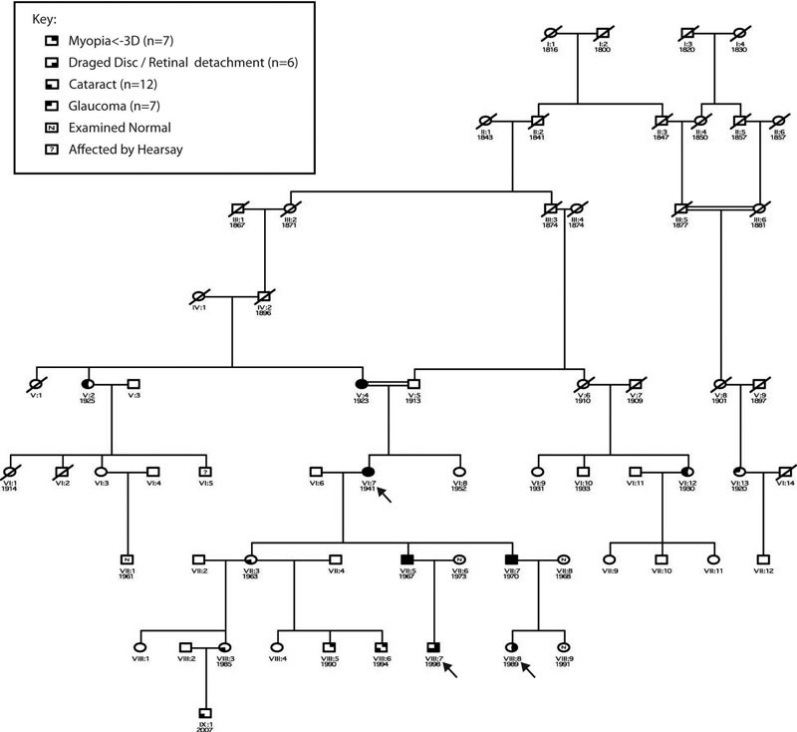
Reduced pedigree showing affected individuals. Square=male, circle=female, Top Right filled=myopia, Bottom Right filled=retinal detachment or dragged disc, Bottom Left filled=cataract, Top Left=primary open-angle glaucoma (POAG), n=examined and normal.
•Table 3 displays the participants’ ophthalmic phenotypes with autorefraction sphere and cylinder, Keratometry readings, and axial length.

•Figure 1 shows the relevant portions of the full pedigree. A consanguineous loop enriched the pedigree with similar genes (RELPAIR [17] analysis suggested a grandparent-grandchild relationship when they were actually great-grandparent and great-grandchild).

**Table 3:** Clinical features of family members examined.

			**Refractive error (D)**		**Keratometry (D)**		**Axial Length (mm)**				
**ID**	**Sex**	**Age at initial examination (years)**	**Right**	**Left**	**Right**	**Left**	**Right**	**Left**	**Cataract**	**Glaucoma**	**Dragged disc or retinal detachment**
V:2	F	81	−0.75/-2.0x70	+0.75/-1.5x65	45.3/44.3	43.8/42.9	25.05	23.56	Yes	Yes	None
V:4	F	83	−2/-3.25x45	−3.25/-1.0x95	49.2/53.9*	48.6/46.2	25.44	24.37	Yes	Yes	Dragged disc
VI:7	F	65	+0.25/-3.0x180	−0.25/-3.5x75	45.3/42.25*	45.8/43.0	23.75	23.88	Yes	Yes	Dragged disc
VI:12	F	76	NR	NR	NR	NR	NR	NR	Yes	Yes	None
VI:13	F	86	NR	NR	NR	NR	NR	NR	No	Yes	None
VII:1	M	45	0/-0.25x180	0/-0.25x180	NR	NR	NR	NR	No	No	None
VII:3	F	43	0/-1.5x180	0/-0.25x160	40.0/43.1	43.4/44.3	NR	NR	Yes	No	None
VII:5	M	39	−3.25/-4.0x180	−0.5/-1x175	43.5/45.1	43.5/43.6	24.60	22.63	Yes	Yes	Dragged disc
VII:6	F	33	−0.75	−0.5/-0.75x170	NR	NR	NR	NR	No	No	None
VII:7	M	36	−2.25/-0.5x155	−6.25/-1.5x145	42.1/ 42.3	43.5/43.1	24.62	26.77	Yes	Yes	Dragged disc
VII:8	F	38	NR	NR	NR	NR	NR	NR	No	No	None
VIII:3	F	25	−0.25/-0.5x88	+0.5/-0.25x102	NR	NR	NR	NR	Yes	No	None
VIII:5	M	16	−1.75/-1.25x50	−6.25/-6.75x175	40.0/43.1	43.4/ 44.3	NR	NR	Yes	No	None
VIII:6	M	12	−2.0/-5.0x90	+0.5/-0.5x160	NR	NR	NR	NR	Yes	No	None
VIII:7	M	8	−5.75/-0.5x55	−9.25/-3.5x95	41.0/41.8	40.0/41.0	25.65	NR	Yes	No	Dragged disc
VIII:8	F	17	+0.5/-7.25x165	ND	NR	ND	NR	ND	ND	ND	Total detachment OU
VIII:9	F	15	−0.25/-0.5x135	0/-0.25x55	NR	NR	NR	NR	No	No	None
IX:1	M	3	1.5	0/-0.5x121	45.3/44.3	43.8/42.9	25.05	NR	Yes	No	None

Abbreviations: F, female; M, male; D, diopters; NR, not recorded; ND, not determinable; OU, both eyes. *measured following cataract surgery
•Figure 2 and Figure 3A-N show photos of the optic disc, retina, and lens.

**Figure 2:**
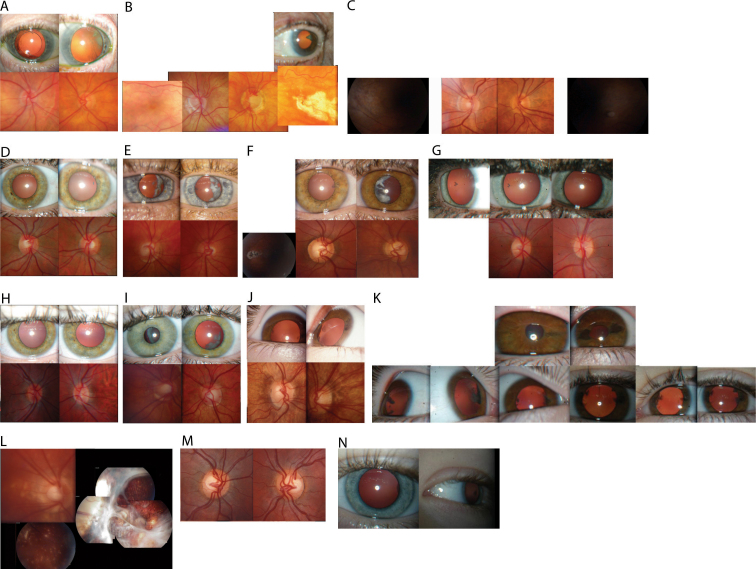
Lens, optic disc, and retina photos of individuals. In the figure, **A** indicates individual V:2; **B** indicates individual V:4; **C** indicates individual VI:7; **D** indicates individual VII:3; **E** indicates individual VII:5; **F** indicates individual VII:7; **G** indicates individual VIII:3; **H** indicates individual VIII:5; **I** indicates individual VIII:6; **J** indicates individual VIII:7; **K** indicates individual VIII:7 followup lens photo five years after first photos; **L** indicates individual VIII:8; **M** indicates individual VIII:9; and **N** indicates individual IX:1.

**Figure 3:**
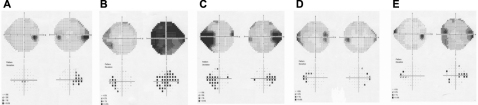
24–2 Humphrey Visual Fields of Individuals. **A** indicates individual V:2; **B** indicates individual V:4; **C** indicates individual VI:7; **D** indicates individual VII:5; and **E** indicates individual VII:7.
•Figure 4A-E show visual field defects.

**Figure 4:**
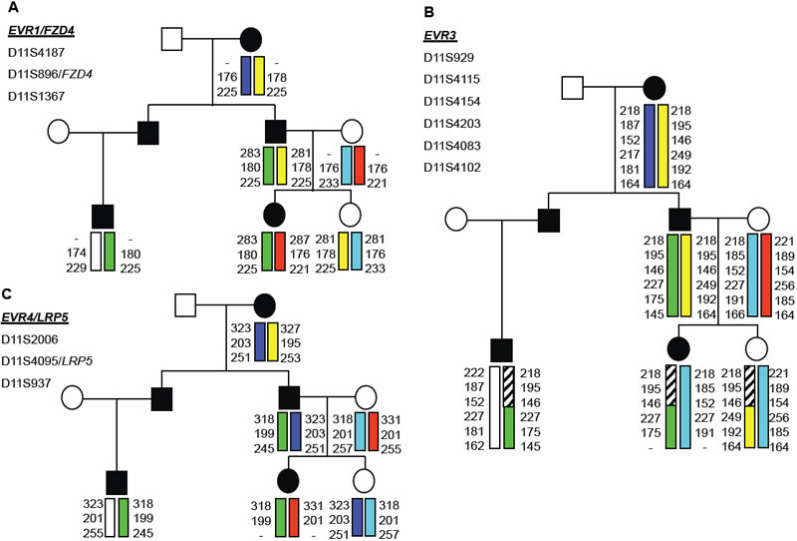
Haplotype analysis of FEVR genes. Only a subset of the pedigree is displayed; shaded individuals are those whose phenotype suggests FEVR. *EVR2* (Norrin) is excluded by the pedigree structure showing male to male transmission. For each locus examined, the affected individuals do not share the same haplotype, indicating that the causative gene does not reside in this region of the chromosomal. **A**: EVR1 (*FZD4*); **B**: EVR3 11p13-p12; **C**: EVR4 (*LRP5*).

Excluding the married-in spouses, we examined eight female and six male family members aged 3–86 years who apparently were affected.

•Visual acuity ranged from 6/5 to perception of light.
•Spherical-equivalent refractive error in Diopters (D) ranged from +0.25 D to −11.0 D, with five individuals having myopia in at least one eye of <-3D.
•Astigmatism varied from 0 to −7.25 D with the rule or −5 D against the rule.
•Axial length varied from 23.75 mm to 26.77 mm.
•Keratometry readings in eyes that had not been operated on ranged from 40.0 D to 48.62 D, with the largest corneal astigmatism measuring only 3.12 D.
•Maximum recorded IOP ranged from 13 mmHg to 36 mmHg.
•Central corneal thickness ranged from 510 μm to 590 μm.
•One male (VIII:6) was found to have a distance exotropia of 25 D.
•Twelve individuals (6 male and 6 female) had CC, thought to be pediatric in onset. (V:2, V:4,VI:7, VI:12, VII:3, VII:5, VII:3, VII:7, VIII:3, VIII:5, VIII:6, VIII:7, IX:1). The youngest age of documented cataract was 3 years of age (IX:1).
•One member (VIII:7) had photographic evidence of cataract progression (Figure 3J,K). In addition, iris atrophy was noted at the 3 and 9 o’clock positions. This atrophy possibly became more notable with age (Figure 3K).
•One female individual (VIII:8) had severe spontaneous retinal detachment consistent with FEVR, while five individuals (3 male and 2 female) had dragged retinal vessels (V:4,VI:7, VII:5, VII:7, VIII:7).
•Seven individuals (5 female and 2 male) had been diagnosed with POAG (V:2, V:4, VI:7, VI:12, VI:13, VII:5, VII:7).

Cataract extraction was performed on VII:7 after the cortical wedge progressed to complete lenticular opacification in the left eye and vision declined from 6/18 to 6/60. Post-operatively, this member’s best-corrected visual acuity improved to 6/6. Refraction in the left eye changed from −6.25/-1.5x145 to +0.00/-0.50 X 98 following cataract surgery. The brother of this individual (VII:5) had similar surgery for cataract and astigmatism, but his visual acuity did not improve from 6/60.

### Systemic associations

None of the family members had dysmorphia or an unusual stature consistent with the facial or body habitus features of Stickler syndrome. One member, who had not worn ear protection in his industrial employment, had noise-related hearing loss (VII:7) and one (V:4) had age-related hearing loss. Only one member (V:4) was found to have a single café-au-lait spot.

One participant (VII:7) had previously been diagnosed with pulmonary alveolar proteinosis (PAP) and treated with repeated pulmonary lavage. PAP is a rare disorder related to the receptor pathway of the granulocyte macrophage–colony stimulating factor (GM-CSF); it was diagnosed after recurrent bouts of pneumonia in adult life. No other family member has experienced similar medical problems; no individual reported any renal problems.

*MYOC* screening of the index case revealed no mutation [14]. Haplotype analysis of a central portion of the pedigree excluded the *EVR1* frizzled family receptor 4 (*FZD4*) and *EVR4* low density lipoprotein receptor-related protein 5 (*LRP5*) *FEVR* genes (Figure 4). Unfortunately, the *EVR3* locus could be only partially excluded due to uninformative markers. Given that this gene had not been identified, we cannot exclude this locus fully. Direct screening of VIII:8 excluded the recently-identified FEVR gene *TSPAN12*.

The family was included in the International High Myopia Consortium linkage analysis [16]; however, the family was dropped from the multipoint analyses for chromosomes 3, 4, 6, 7, 8, 11, and 12 due to the pedigree’s complexity. Table 4 displays the two-point linkage results for this family showing the highest scoring logarithm of odds (LOD) scores above 1.5. There were multiple chromosomal SNPs of interest, but no statistically significant focal localization.

**Table 4:** Summary of the Johns Hopkins Center for Inherited Disease Research (CIDR) results for the family.

**Chromosome**	**Marker**	**Position (cM)**	**2PT-parametric (Fastlink)**	**MPT-non-parametric**	**MPT-parametric**
1	rs1981193	121.82	*1.863*	NS	NS
1	rs1806753	160.34	*1.079*	NS	NS
2	rs2053372	47.98	*1.592*	NS	NS
2	rs2008535	54.9	*1.128*	NS	NS
2	rs764464	65.31	*1.328*	NS	NS
2	rs1022298	117.27	*1.162*	NS	NS
2	rs264963	117.39	*1.162*	NS	NS
3	rs2076993	46.5	*1.166*	NS	NS
3	rs1348979	49.44	*1.166*	NS	NS
3	rs1127732	59.51	*1.097*	NS	NS
3	rs713144	60.4	*1.477*	NS	NS
3	rs1382554	60.41	*1.097*	NS	NS
3	rs1405793	64.61	*1.159*	NS	NS
3	rs1495704	65.68	*1.159*	NS	NS
3	rs1995137	66.29	*1.159*	NS	NS
3	rs1351631	67.73	*1.522*	NS	NS
3	rs737516	67.73	*1.522*	NS	NS
3	rs1013758	67.81	*1.522*	NS	NS
3	rs844438	78.91	*1.123*	NS	NS
3	rs1447971	82.11	*1.842*	NS	NS
3	rs935734	92.98	*1.586*	NS	NS
3	rs1019374	95	*1.069*	NS	NS
3	rs1388276	99.96	*1.116*	NS	NS
4	rs751266	67.19	*1.054*	NS	NS
4	rs896656	93.96	*1.326*	NS	NS
8	rs2203837	23.58	*1.615*	NS	NS
8	rs334206	32.33	*1.241*	NS	NS
8	rs241202	48.58	*1.849*	NS	NS
8	rs4107736	50.87	*1.248*	NS	NS
8	rs1481747	53.13	*1.103*	NS	NS
8	rs1955185	61.16	*1.05*	NS	NS
8	rs716583	65.56	*1.116*	NS	NS
8	rs344278	74.88	*1.582*	NS	NS
8	rs1460239	112.26	*1.618*	NS	NS
8	rs1433396	122.14	*1.119*	NS	NS
8	rs766811	138.68	*1.16*	NS	NS
9	rs1532310	0.124137	*1.522*	NS	NS
9	rs1532309	0.124434	*1.522*	NS	NS
9	rs1143025	30.9	*1.176*	NS	NS
9	rs1029015	35.12	*1.767*	NS	NS
9	rs716933	60.37	*1.089*	NS	NS
9	rs987187	60.4	*1.128*	NS	NS
9	rs1333342	69.96	*1.477*	NS	NS
10	rs1346300	75.86	*1.522*	NS	NS
11	rs676943	125.79	*1.015*	NS	NS
12	rs871880	58.31	*1.123*	NS	NS
12	rs7134835	161.7	*1.2*	NS	NS
12	rs1278602	171.56	*1.089*	NS	NS
12	rs1278601	171.57	*1.089*	NS	NS
12	rs937538	171.78	*1.094*	NS	NS
13	rs2985981	49.25	*1.004*	NS	NS
13	rs2031836	115.73	*1.003*	NS	NS
15	rs1435735	46.31	*1.199*	NS	NS
15	rs890153	46.31	*1.554*	NS	NS
15	rs725463	60.22	*1.043*	NS	NS
15	rs1445020	71.05	*1.049*	NS	NS
16	rs1019141	19.98	*1.49*	NS	NS
16	rs889593	122.83	0.018	*0.701998*	1.0217
16	rs299956	123.93	0.734	*0.943619*	1.5971
16	rs2076962	125.29	−0.036	*1.127055*	1.8771
16	rs3794668	126.97	−0.011	*1.126755*	1.8763
16	rs1056707	128.94	0.057	*1.12803*	1.8782
16	rs750740	129.03	0.399	*1.128125*	1.8783
16	rs463701	130.14	−0.067	*1.129806*	1.8804
16	rs452176	130.21	0.01	*1.129825*	1.8804
16	rs1006547	130.48	0.018	*1.129924*	1.8805
16	rs1800330	130.5	0.891	NS	NS
**16**	**rs870856**	**130.83**	***1.781***	***1.126244***	**1.8762**
16	rs8577	130.86	0.549	*1.125715*	1.8755
17	rs721429	95.95	*1.199*	NS	NS
18	rs1972602	45.77	*1.123*	NS	NS
18	rs1548755	51.57	*1.252*	NS	NS
18	rs1131709	56.82	*1.339*	NS	NS
18	rs650680	58.25	*1.767*	NS	NS
18	rs931078	84.57	*1.11*	NS	NS
20	rs1535382	14.16	*1.046*	NS	NS
21	rs1041756	33.98	*1.07*	NS	NS
21	rs2839576	62.26	*1.324*	NS	NS

2-point analyses with Fastlink under a dominant model; multipoint results, both parametric and non-parametric, using the multipoint engine for rapid likelihood inference (MERLIN). Results in italics highlight suggestive loci, while the results in bold were found to be suggestive under all models tested. Abbreviations: Chr, chromosome; cM, centimorgan; 2PT, two point; MPT, multi-point; NS, not significant.

## Discussion

This Australian pedigree has a unique constellation of ophthalmic features that do not appear to have been described previously. Although we were unable to identify a similar family reported in the literature, the subtle and relatively common clinical features could be overlooked.

Many investigators have reported the association of high myopia with ocular morbidities of early-onset cataract, glaucoma and retinal detachment [5]. Pedigrees with myopia are common, but pedigrees with so many members affected with these early ocular issues along with myopic development are extremely rare; we were not able to identify any in the published literature.

Although we cannot discount that the associated ocular features may be secondary in origin, this family raises the possibility that the same gene may be responsible for all forms of the pathology observed in the pedigree.

Retinal detachment is an uncommon disorder in young people and is most commonly identified in patients with FEVR. X-linked FEVR and Norrie disease arose from mutations in Norrin (excluded by male-to-male transmission, in this pedigree). Dominant FEVR is due to mutations in *FZD4* and *LRP5,* and has been linked to the *EVR3* locus [18]. We excluded these loci through linkage analysis. The recently-described gene *TSPAN12* (*EVR5*) was excluded by sequence analysis. Nonetheless, despite a well characterized FEVR mutation, there still can be considerable variation in the expressivity of the phenotype and incomplete penetrance [15,18,19] (Personal communication; T.L. Edwards, Centre for Eye Research Australia, Melbourne, Australia [article in press]).

Since the cataract is the most “easily characterized” phenotype in this family’s pedigree, we compared it with other cataract phenotypes described in the literature. Although CC has been linked to or associated with many cataract loci and many chromosomal deletions, the causative mutation has not been identified for the majority of CC and pediatric cataract cases [6].

The peripheral cortical lamella wedge seen in this family is similar to that observed in Stickler syndrome [7] and also with neurofibromatosis Type 2 (NF2) [20]. Interestingly, one case describes NF2 associated with posterior subcapsular cataract and dragged disc [21]. In a series of 15 other NF2 patients, 12 patients had an epiretinal membrane in the macular or paramacular area and 11 patients had central posterior cortical, subcapsular, or peripheral cortical lens opacities [22]. NF2 arises from mutations in the *Merlin* gene on chromosome 22q12.2 [23].

The one case of PAP [24] prompted an investigation of possible genes involved in the GM-CSF pathway using the Online Mendelian Inheritance in Man^®^ (OMIM) database at Johns Hopkins University. Of three loci associated with PAP, one gene located at chromosome 22q12.2-q13.1, Granulocyte-macrophage Colony-stimulating factor receptor, beta (*CSF2RB*) is adjacent to *Merlin*. Notably, on reviewing myopia loci, the myopia linkage found by Stambolian and colleagues [25] for marker D22S685 lies in chromosome region 22q12. This region has also been replicated in the Beaver Dam Eye study [26].

The refractive error recorded in this pedigree is atypical; most hereditary myopia is symmetric and usually is not associated with high astigmatism. To date there has been little investigation of the genetics of astigmatism, though genetic factors are likely to play a role [27]. It would appear that the myopia in this family originates in increased axial length rather than in the more usual primary lenticular fault. The degree of astigmatism in severely affected members, however, appeared to be both lenticular and corneal, suggesting a common mechanism of growth or compensation. The causative interaction of the cataract and the increased myopia remains to be elucidated, but may involve visual form deprivation [28].

We hope that characterization of this unusual phenotypic constellation will identify other families with similar characteristics. Further characterization of the genes involved in this family using methods such as next-generation sequencing should help shed light on the genetics of the four clinical entities —POAG, CC, FEVR, and myopia— as well as their interactions. In time, this further work also may help clarify the molecular pathways of developing myopia involving retinal signaling, lens growth and axial length.
